# Data relating to spatial distribution of polycyclic aromatic hydrocarbons in terrestrial soils of Pakistan and King George Island, Antarctica

**DOI:** 10.1016/j.dib.2019.104327

**Published:** 2019-08-08

**Authors:** Siwatt Pongpiachan, Woranuch Deelaman, Chomsri Choochuay, Natthapong Iadtem, Vanisa Surapipith, Muhammad Zaffar Hashmi, Muhammad Latif, Muhammad Sohail, Syed Ali Musstjab Akber Shah Eqani, Teetat Charoenkalunyuta, Kittiphop Promdee

**Affiliations:** a NIDA Center for Research & Development of Disaster Prevention & Management, School of Social and Environmental Development, National Institute of Development Administration (NIDA, 118 Moo 3, Sereethai Road, Klong-Chan, Bangkapi, Bangkok, 10240, Thailand; b Faculty of Environmental Management, Prince of Songkla University, Hat-Yai, Songkla, 90112, Thailand; c Department of Meteorology, COMSATS University, Park Road, Chak Shahzad, Islamabad, 44000, Pakistan; d Department of Bioscience, COMSATS University, Park Road, Chak Shahzad, Islamabad, 44000, Pakistan; e Department of Environmental Science, Chulachomklao Royal Military Academy, Nakhon Nayok, 26001, Thailand; f National Astronomical Research Institute of Thailand (Public Organization), 260 Moo 4, T. Donkaew, A. Maerim, Chiang-Mai, 50180, Thailand; g Department of Survey Engineering, Chulalongkorn University, Bangkok, 10330, Thailand

**Keywords:** Polycyclic aromatic hydrocarbons (PAHs), Terrestrial soils, Pakistan, King george island, Antarctica

## Abstract

Over the past few decades, polycyclic aromatic hydrocarbons (PAHs) have been analysed in various environmental compartments, however, only limited information is available associated with their terrestrial concentrations in Pakistan and Antarctica. All terrestrial soils from Pakistan (n = 120) were collected from 14th to 2nd April 2017 at Islamabad (*n* = 30), Abbotabad (*n* = 10), Taxilla (*n* = 5), and other places from north to south (*n* = 75). All Antarctic terrestrial soils (*n* = 11) were collected from 1st to 25th February 2018 in the southwestern part of King George Island. It is crucial to underline that all samples were both qualitatively and quantitatively identified by using a Shimadzu GCMS-QP2010 Ultra system coupled with a high-speed performance system with ASSP function (i.e., achieving maximum scan speed of 20,000 u sec^−1^) and having ultra-fast data acquisition speed for comprehensive two-dimensional gas chromatography (GC × GC). Analytical results implicate the influences of vehicle exhausts as a major contributor of PAHs in terrestrial soils of Pakistan. It seems rationale to conclude that 3-ring PAHs display the majority of PAH congeners in terrestrial soils of King George Island.

**Table:** Specifications Table

Subject area	Environmental Sciences
More specific subject area	Environmental Chemistry
Type of data	Table, text file, graph, figure
How data was acquired	Soxhlet extraction and Shimadzu GCMS-QP2010 Ultra system coupled with a high-speed performance system with ASSP function [1]
Data format	Raw data, analysed.
Experimental factors	Sampling protocol precautions, sample preparation, and quality control/quality assurance (QA/QC) were comprehensively documented in United States Environmental Protection Agency (US-EPA) Method 5035 for soil sampling and will not be mentioned here [2]. Chemical extraction is performed in Soxhlet equipment with dichloromethane and hexane [3].
Experimental features	PAH congeners applying GC-MS.
Data source locations	All samples were collected from terrestrial soils located at Hunza, Gilgit, Skardu, Kalam Swat, Gulibagh Swat, Malamjaba Swat, Swabi, Nowshera, Mianwali, Bhakkar, Layyah, D.G Khan, Sukkar, Khairpur, Hyderabad, Abbottabad, Mansehra, Murree, Islamabad, and King George Island.
Data accessibility	Data available within article.

**Table:** 

**Value of the data** • PAHs with low biodegradability and high persistency in environment, which is acknowledged as priority pollutants by US EPA as a consequence of its carcinogenic and mutagenic impacts therefore applying the suitable policy is the requirement to control PAHs and reducing of its concern. Data can be used as to facilitate policy and decision making process in order to control and decreasing the level of PAH contamination present in the terrestrial soils of Pakistan and King George Island, Antarctica. • Since long range atmospheric transportation is responsible for POPs contamination of pristine and sensitive environments, a long term monitoring of PAH congeners is therefore essentially crucial for conducting environmental risk assessment at King George Island, Antarctica. • Data exhibited here may serve as benchmarks for other scientific communities focusing in the field of ecological toxicology to evaluate human expose to PAHs via dietary, inhalation, and dermal contact in Pakistan and King George Island, Antarctica. • The present data offers detailed information on molecular fingerprints of soil PAHs as obtained through GC/MS-MS. Further investigations for source identifications can be conducted by using diagnostic binary ratios of PAHs provided by this study. • Data set of PAHs collected at King George Island, Antarctica can be used to conduct the source apportionment (e.g. principal component analysis (PCA), positive matrix factorization (PMF), and UNMIX) of PAHs in terrestrial soils of Pakistan and King George Island.

## 1 Data

Table 1 and Table 2 demonstrate sampling positions of terrestrial soil samples collected from Pakistan and King George Island, respectively. Table 3, Table 4 and Table 5, Table 6 are presenting the concentrations of PAHs collected at Pakistan and King George Island, respectively.

**Table 1:** Sampling positions of terrestrial soils in Pakistan.

No.	Latitude		Longitude	
I1	33.6997	N	73.0092	E
I2	33.7192	N	73.0464	E
I3	33.7042	N	73.0578	E
I4	33.6978	N	73.0617	E
I5	33.6922	N	73.0664	E
I6	33.6803	N	73.0758	E
I7	33.6628	N	73.0842	E
I8	33.6656	N	73.0867	E
I9	33.6564	N	73.0942	E
I10	33.6575	N	73.0928	E
I11	33.6439	N	73.1028	E
I12	33.6364	N	73.1086	E
I13	33.6272	N	73.1153	E
I14	33.6206	N	73.1203	E
I15	33.6114	N	73.1272	E
I16	33.6028	N	73.1336	E
I17	33.5967	N	73.1381	E
I18	33.5944	N	73.1378	E
I19	33.5828	N	73.1486	E
I20	33.5719	N	73.1564	E
I21	33.5669	N	73.1603	E
I22	33.5589	N	73.1664	E
I23	33.5517	N	73.1717	E
I24	33.5411	N	73.1794	E
I25	33.5344	N	73.1811	E
I26	33.5292	N	73.1806	E
I27	33.5183	N	73.1794	E
I28	33.5078	N	73.1833	E
I29	33.4886	N	73.1972	E
I30	33.4686	N	73.1997	E
AB1	34.2006	N	73.2383	E
AB2	34.1844	N	73.2317	E
AB3	34.1792	N	73.2289	E
AB4	34.1711	N	73.2256	E
AB5	34.1961	N	73.2342	E
AB6	34.1997	N	73.2378	E
AB7	34.1225	N	73.1853	E
AB8	34.1083	N	73.1722	E
AB9	34.0558	N	73.1492	E
AB10	34.0228	N	73.1058	E
TA1	34.7039	N	72.8244	E
TA2	33.7458	N	72.8183	E
TA3	33.7692	N	72.8642	E
TA4	33.7686	N	72.8633	E
TA5	33.7114	N	72.8150	E
SA-TOP	34.9200	N	73.1300	E
SA-10	34.9200	N	73.1300	E
SA-20	34.9200	N	73.1300	E
SA-30	34.9200	N	73.1300	E
SB-TOP	34.2000	N	73.1200	E
SB-10	34.2000	N	73.1200	E
SB-20	34.2000	N	73.1200	E
SB-30	34.2000	N	73.1200	E
SC-TOP	33.9000	N	73.3900	E
SC-10	33.9000	N	73.3900	E
SC-20	33.9000	N	73.3900	E
SC-30	33.9000	N	73.3900	E
SD-TOP	33.4300	N	73.0400	E
SD-10	33.4300	N	73.0400	E
SD-20	33.4300	N	73.0400	E
SD-30	33.4300	N	73.0400	E
S1-TOP	36.3167	N	74.6500	E
S1-10	36.3167	N	74.6500	E
S1-20	36.3167	N	74.6500	E
S1-30	36.3167	N	74.6500	E
S2-TOP	35.9219	N	74.2892	E
S2-10	35.9219	N	74.2892	E
S2-20	35.9219	N	74.2892	E
S2-30	35.9219	N	74.2892	E
S3-TOP	35.3000	N	75.6167	E
S3-10	35.3000	N	75.6167	E
S3-20	35.3000	N	75.6167	E
S3-30	35.3000	N	75.6167	E
S4-TOP	35.3833	N	72.1833	E
S4-10	35.3833	N	72.1833	E
S4-20	35.3833	N	72.1833	E
S4-30	35.3833	N	72.1833	E
S5-TOP	35.3700	N	72.2130	E
S5-10	35.3700	N	72.2130	E
S5-20	35.3700	N	72.2130	E
S5-30	35.3700	N	72.2130	E
S6-TOP	35.3300	N	72.1130	E
S6-10	35.3300	N	72.1130	E
S6-20	35.3300	N	72.1130	E
S6-30	35.3300	N	72.1130	E
S7-TOP	34.1167	N	72.4667	E
S7-10	34.1167	N	72.4667	E
S7-20	34.1167	N	72.4667	E
S7-30	34.1167	N	72.4667	E
S8-TOP	34.0153	N	71.9747	E
S8-10	34.0153	N	71.9747	E
S8-20	34.0153	N	71.9747	E
S8-30	34.0153	N	71.9747	E
S9-TOP	32.5854	N	71.5436	E
S9-10	32.5854	N	71.5436	E
S9-20	32.5854	N	71.5436	E
S9-30	32.5854	N	71.5436	E
S10-TOP	31.6333	N	71.0667	E
S10-20	31.6333	N	71.0667	E
S10-30	31.6333	N	71.0667	E
S11-TOP	30.9602	N	70.9423	E
S11-10	30.9602	N	70.9423	E
S11-20	30.9602	N	70.9423	E
S11-30	30.9602	N	70.9423	E
S12-TOP	30.0500	N	70.6333	E
S12-10	30.0500	N	70.6333	E
S12-20	30.0500	N	70.6333	E
S12-30	30.0500	N	70.6333	E
S13-TOP	27.7056	N	68.8472	E
S13-10	27.7056	N	68.8472	E
S13-20	27.7056	N	68.8472	E
S13-30	27.7056	N	68.8472	E
S14-TOP	27.3200	N	68.4600	E
S14-10	27.3200	N	68.4600	E
S14-20	27.3200	N	68.4600	E
S14-30	27.3200	N	68.4600	E
S15-TOP	25.3800	N	68.3700	E
S15-10	25.3800	N	68.3700	E
S15-20	25.3800	N	68.3700	E
S15-30	25.3800	N	68.3700	E

**Table 2:** Sampling positions of terrestrial soils in King George Island, sub Antarctica.

No.	Latitude		Longitude	
1	62.209667	S	58.967972	W
2	62.209583	S	58.967333	W
3	62.226278	S	58.950583	W
4	62.207667	S	58.959361	W
5	62.211389	S	58.935222	W
6	62.218639	S	58.971972	W
7	62.195611	S	58.970972	W
8	62.190972	S	58.974250	W
9	62.196500	S	58.993306	W
10	62.208083	S	58.960750	W
11	62.207778	S	58.959167	W

**Table 3:** PAH congener concentrations (pg g^−1^) of Phe, An, Fluo, Pyr, 11H-B[a]F, 11H-B[b]F, and B[a]A in terrestrial soils of Pakistan.

	Phe	An	Fluo	Pyr	11H-B[a]F	11H-B[b]F	B[a]A
IS1	2.66E+03	3.23E+02	1.27E+03	1.16E+03	2.87E+02	1.16E+02	4.11E+02
IS2	9.75E+02	1.36E+02	6.70E+02	6.15E+02	2.67E+02	1.57E+02	3.16E+02
IS3	2.94E+03	3.64E+02	1.26E+03	1.13E+03	3.52E+02	1.28E+02	2.85E+02
IS4	3.48E+03	4.95E+02	2.42E+03	2.06E+03	4.75E+02	1.07E+02	7.57E+02
IS5	1.74E+03	1.80E+02	1.33E+03	1.08E+03	1.49E+02	3.71E+01	3.65E+02
IS6	1.01E+03	6.91E+01	7.87E+02	7.87E+02	1.22E+02	5.20E+01	1.80E+02
IS7	8.81E+02	8.87E+01	7.77E+02	6.02E+02	6.61E+02	4.27E+02	1.56E+02
IS8	1.32E+03	1.32E+02	1.58E+03	1.66E+03	9.69E+02	5.60E+02	4.71E+02
IS9	1.41E+03	1.73E+02	1.89E+03	1.48E+03	7.15E+02	3.66E+02	5.77E+02
IS10	1.81E+03	2.04E+02	1.61E+03	1.49E+03	9.80E+02	5.40E+02	5.80E+02
IS11	1.60E+03	1.45E+02	1.38E+03	1.59E+03	2.58E+02	9.22E+01	4.30E+02
IS12	1.20E+03	1.52E+02	8.90E+02	9.26E+02	1.47E+02	2.19E+01	1.43E+02
IS13	8.01E+02	8.29E+01	6.00E+02	4.38E+02	9.80E+01	3.08E+01	1.05E+02
IS14	5.36E+03	3.69E+02	1.87E+03	1.66E+03	3.34E+02	4.89E+01	3.52E+02
IS15	9.13E+02	9.38E+01	5.11E+02	4.45E+02	5.06E+01	8.85E+00	8.96E+01
IS16	1.45E+03	7.60E+01	5.45E+02	7.56E+02	7.97E+01	4.39E+01	8.05E+01
IS17	1.62E+03	1.70E+02	1.42E+03	1.02E+03	2.02E+02	3.53E+01	3.08E+02
IS18	1.53E+03	1.92E+02	1.17E+03	9.38E+02	1.89E+02	5.58E+01	3.01E+02
IS19	1.01E+03	9.78E+01	3.91E+02	2.63E+02	6.70E+01	1.08E+01	5.59E+01
IS20	5.19E+02	6.76E+01	2.65E+02	1.96E+02	5.08E+01	1.12E+01	4.11E+01
IS21	1.60E+03	1.85E+02	8.73E+02	7.48E+02	1.39E+02	3.47E+01	1.64E+02
IS22	1.22E+03	1.41E+02	8.03E+02	6.04E+02	1.26E+02	2.01E+01	1.77E+02
IS23	6.52E+02	8.13E+01	3.00E+02	2.15E+02	4.78E+01	1.04E+01	4.71E+01
IS24	2.56E+03	2.60E+02	3.49E+03	3.06E+03	5.31E+02	1.11E+02	1.16E+03
IS25	6.80E+02	8.22E+01	3.36E+02	2.92E+02	3.59E+01	1.76E+01	7.44E+01
IS26	9.33E+02	1.57E+02	5.97E+02	4.93E+02	1.00E+02	1.72E+01	1.71E+02
IS27	5.17E+02	5.14E+01	1.71E+02	1.30E+02	1.26E+01	5.21E+00	1.92E+01
IS28	3.96E+03	7.12E+02	1.50E+04	1.22E+04	1.56E+03	1.87E+02	3.36E+03
IS29	5.54E+03	6.03E+02	3.07E+03	2.30E+03	3.47E+02	5.94E+01	6.21E+02
IS30	2.01E+03	1.83E+02	8.35E+02	7.19E+02	1.21E+02	3.24E+01	1.14E+02
AB1	3.20E+05	1.13E+05	4.31E+05	3.84E+05	1.74E+05	1.33E+05	3.42E+05
AB2	4.18E+03	6.86E+02	8.00E+03	6.42E+03	1.34E+03	4.15E+02	3.13E+03
AB3	6.38E+03	6.76E+02	6.38E+03	3.79E+03	6.80E+02	2.70E+02	9.22E+02
AB4	8.38E+03	1.50E+03	1.10E+04	1.05E+04	1.94E+03	6.70E+02	4.29E+03
AB5	4.81E+04	8.82E+03	7.38E+04	6.17E+04	1.09E+04	5.39E+03	2.78E+04
AB6	1.86E+03	2.35E+02	2.21E+03	1.90E+03	4.73E+02	8.60E+01	6.60E+02
AB7	2.46E+03	4.41E+02	2.81E+03	2.27E+03	8.14E+02	1.14E+03	1.30E+03
AB8	5.47E+03	8.24E+02	8.34E+03	7.07E+03	1.43E+03	3.43E+02	2.64E+03
AB9	2.57E+03	4.60E+02	4.74E+03	3.84E+03	5.88E+02	1.09E+02	1.46E+03
AB10	1.09E+04	1.63E+03	3.64E+04	2.92E+04	4.20E+03	9.30E+02	1.23E+04
TA1	3.75E+03	3.77E+02	3.12E+03	2.65E+03	4.92E+02	1.00E+02	1.14E+03
TA2	8.02E+03	1.82E+03	3.86E+04	3.22E+04	3.69E+03	3.03E+02	1.35E+04
TA3	8.02E+03	1.82E+03	3.86E+04	3.22E+04	3.69E+03	3.03E+02	1.35E+04
TA4	1.10E+04	6.30E+02	1.66E+04	1.49E+04	1.39E+03	6.26E+01	4.21E+03
TA5	1.06E+04	2.76E+03	4.67E+04	3.90E+04	5.23E+03	5.98E+02	2.33E+04
SA-TOP	3.23E+03	3.59E+02	2.60E+03	2.46E+03	8.26E+02	1.88E+02	1.73E+03
SA-10	5.93E+03	5.74E+02	4.87E+03	4.70E+03	1.45E+03	2.59E+02	2.06E+03
SA-20	1.96E+03	2.17E+02	2.15E+03	1.87E+03	3.76E+02	8.84E+01	7.20E+02
SA-30	3.28E+03	3.53E+02	3.05E+03	2.68E+03	8.14E+02	1.62E+02	1.28E+03
SB-TOP	2.27E+03	1.44E+02	7.12E+02	5.55E+02	1.32E+02	3.09E+01	1.32E+02
SB-10	2.55E+03	1.63E+02	1.06E+03	8.01E+02	2.25E+02	2.10E+01	1.23E+02
SB-20	1.79E+03	1.47E+02	6.34E+02	4.81E+02	1.09E+02	1.71E+01	6.29E+01
SB-30	1.68E+03	1.07E+02	7.09E+02	4.66E+02	9.54E+01	1.11E+01	6.10E+01
SC-TOP	2.65E+03	2.99E+02	2.18E+03	1.84E+03	5.19E+02	1.12E+02	8.49E+02
SC-10	3.77E+03	3.89E+02	4.94E+03	4.13E+03	9.23E+02	2.28E+02	1.99E+03
SC-20	1.29E+03	7.55E+01	4.18E+02	3.22E+02	8.66E+01	1.19E+01	3.55E+01
SC-30	2.01E+03	1.50E+02	4.76E+02	3.27E+02	6.07E+01	1.05E+01	4.22E+01
SD-TOP	1.80E+03	1.10E+02	1.29E+03	9.71E+02	1.46E+02	1.48E+01	1.63E+02
SD-10	2.20E+03	1.41E+02	1.46E+03	1.23E+03	1.98E+02	3.54E+01	3.07E+02
SD-20	3.29E+03	1.99E+02	1.95E+03	1.96E+03	4.42E+02	1.66E+02	9.26E+02
SD-30	1.78E+03	9.86E+01	7.69E+02	6.16E+02	1.20E+02	2.06E+01	1.10E+02
S1-TOP	2.15E+03	9.07E+01	8.11E+02	7.51E+02	2.69E+02	2.81E+01	1.93E+02
S1-10	2.08E+03	8.76E+01	5.83E+02	4.46E+02	1.18E+02	2.47E+01	8.14E+01
S1-20	2.77E+03	1.39E+02	1.08E+03	8.11E+02	1.52E+02	4.83E+01	1.41E+02
S1-30	3.68E+03	1.34E+02	1.11E+03	8.38E+02	2.05E+02	3.98E+01	2.34E+02
S2-TOP	4.06E+03	1.90E+02	1.63E+03	1.17E+03	1.96E+02	4.24E+01	1.97E+02
S2-10	3.43E+03	1.87E+02	1.67E+03	1.49E+03	2.39E+02	4.14E+01	3.41E+02
S2-20	3.03E+03	1.34E+02	1.12E+03	9.12E+02	1.77E+02	3.77E+01	2.52E+02
S2-30	3.45E+03	1.97E+02	1.17E+03	9.56E+02	1.50E+02	3.33E+01	1.18E+02
S3-TOP	2.69E+03	1.06E+02	7.39E+02	6.13E+02	8.96E+01	2.23E+01	6.22E+01
S3-10	1.68E+03	7.52E+01	4.70E+02	3.64E+02	6.30E+01	1.42E+01	2.62E+01
S3-20	1.89E+03	7.38E+01	4.91E+02	4.03E+02	6.21E+01	2.23E+01	2.33E+01
S3-30	1.45E+03	7.62E+01	4.30E+02	4.13E+02	5.47E+01	1.09E+01	2.76E+01
S4-TOP	4.55E+03	3.15E+02	1.87E+03	1.65E+03	3.07E+02	7.77E+01	6.13E+02
S4-10	3.00E+03	2.36E+02	1.57E+03	1.52E+03	3.05E+02	6.73E+01	6.44E+02
S4-20	6.30E+03	5.23E+02	2.05E+03	1.81E+03	3.03E+02	5.90E+01	4.02E+02
S4-30	2.91E+03	1.65E+02	1.27E+03	1.05E+03	2.00E+02	4.42E+01	3.47E+02
S5-TOP	2.36E+03	1.19E+02	6.35E+02	4.68E+02	8.59E+01	1.82E+01	3.74E+01
S5-10	3.32E+03	2.97E+02	2.05E+03	1.92E+03	4.24E+02	6.67E+01	7.90E+02
S5-20	2.29E+03	1.15E+02	6.15E+02	4.54E+02	8.32E+01	1.76E+01	3.62E+01
S5-30	2.79E+03	2.38E+02	1.42E+03	1.39E+03	3.10E+02	3.05E+01	5.23E+02
S6-TOP	2.79E+03	2.62E+02	1.25E+03	1.01E+03	2.36E+02	2.99E+01	3.26E+02
S6-10	2.83E+03	1.53E+02	9.05E+02	7.63E+02	1.76E+02	2.06E+01	4.74E+01
S6-20	8.57E+03	4.64E+02	1.65E+03	1.35E+03	1.66E+02	3.85E+01	6.40E+01
S6-30	2.35E+03	1.32E+02	5.45E+02	5.23E+02	7.49E+01	6.92E+00	3.55E+01
S7-TOP	1.97E+03	1.99E+02	1.08E+03	9.81E+02	1.60E+02	2.97E+01	2.45E+02
S7-10	1.74E+03	1.66E+02	7.82E+02	7.23E+02	1.55E+02	2.51E+01	1.91E+02
S7-20	2.37E+03	2.50E+02	3.35E+03	2.90E+03	5.24E+02	1.38E+02	1.70E+03
S7-30	1.23E+03	1.38E+02	5.01E+02	4.84E+02	1.01E+02	8.25E+00	7.75E+01
S8-TOP	3.59E+03	3.47E+02	2.04E+03	1.81E+03	2.44E+02	4.14E+01	4.28E+02
S8-10	1.85E+03	1.89E+02	1.99E+03	1.78E+03	2.41E+02	2.80E+01	5.25E+02
S8-20	1.91E+03	1.91E+02	2.07E+03	1.92E+03	2.63E+02	2.97E+01	5.52E+02
S8-30	6.11E+03	1.99E+02	1.74E+03	1.74E+03	3.27E+02	7.45E+01	6.62E+02
S9-TOP	4.98E+03	5.49E+02	2.01E+03	1.56E+03	2.79E+02	4.64E+01	2.84E+02
S9-10	2.33E+03	1.87E+02	1.05E+03	8.49E+02	1.90E+02	3.01E+01	2.30E+02
S9-20	4.57E+03	3.91E+02	1.72E+03	1.44E+03	2.62E+02	5.30E+01	3.27E+02
S9-30	4.67E+03	2.20E+02	2.13E+03	1.61E+03	2.53E+02	4.03E+01	4.28E+02
S10-TOP	1.34E+03	8.49E+01	7.06E+02	5.38E+02	1.26E+02	1.90E+01	1.24E+02
S10-20	2.47E+03	7.97E+01	6.80E+02	7.09E+02	6.42E+01	1.00E+01	4.60E+01
S10-30	2.26E+03	9.97E+01	5.48E+02	4.12E+02	6.36E+01	9.14E+00	2.75E+01
S11-TOP	3.80E+03	3.91E+02	2.59E+03	2.01E+03	4.34E+02	7.91E+01	1.19E+03
S11-10	4.20E+03	2.63E+02	1.08E+03	7.57E+02	1.57E+02	4.15E+01	9.24E+01
S11-20	4.04E+03	2.49E+02	1.05E+03	7.26E+02	1.27E+02	2.68E+01	6.00E+01
S11-30	2.37E+03	1.62E+02	6.89E+02	5.11E+02	1.13E+02	1.54E+01	6.88E+01
S12-TOP	2.05E+03	1.29E+02	4.65E+02	3.01E+02	0.00E+00	0.00E+00	1.01E+02
S12-10	9.94E+02	1.27E+02	9.40E+02	1.21E+03	3.30E+02	5.21E+01	5.87E+02
S12-20	1.95E+03	1.04E+02	7.43E+02	6.10E+02	2.51E+02	1.42E+01	1.24E+02
S12-30	2.73E+03	2.50E+02	5.65E+02	4.42E+02	7.28E+01	1.43E+01	8.36E+01
S13-TOP	3.56E+03	3.73E+02	1.23E+03	9.37E+02	1.12E+02	2.73E+01	9.99E+01
S13-10	2.58E+03	2.95E+02	7.68E+02	5.84E+02	9.86E+01	1.57E+01	9.37E+01
S13-20	1.98E+03	1.83E+02	1.93E+03	1.68E+03	2.46E+02	4.57E+01	5.14E+02
S13-30	2.22E+03	2.05E+02	8.11E+02	6.19E+02	1.33E+02	1.15E+01	7.07E+01
S14-TOP	1.39E+03	1.04E+02	9.83E+02	8.96E+02	1.73E+02	2.05E+01	1.91E+02
S14-10	1.84E+03	1.96E+02	9.56E+02	7.88E+02	1.17E+02	1.62E+01	9.66E+01
S14-20	2.21E+03	2.04E+02	8.09E+02	6.18E+02	1.33E+02	1.15E+01	7.05E+01
S14-30	7.04E+02	5.27E+01	2.91E+02	2.67E+02	4.93E+01	1.13E+01	4.47E+01
S15-TOP	1.54E+03	1.82E+02	2.72E+03	2.08E+03	3.05E+02	2.49E+01	8.70E+02
S15-10	1.26E+03	1.12E+02	7.02E+02	6.65E+02	1.17E+02	3.03E+01	1.86E+02
S15-20	1.10E+03	6.18E+01	7.53E+02	7.18E+02	1.02E+03	1.13E+02	5.37E+02
S15-30	1.20E+03	1.27E+02	6.07E+02	5.36E+02	1.58E+02	3.34E+01	1.94E+02

**Table 4:** PAH congener concentrations (pg g^−1^) of Chry, B[b]F, B[k]F, B[e]P, B[a]P, Ind, D[a,h]A, and B[g,h,i]P in terrestrial soils of Pakistan.

	Chry	B[b]F	B[k]F	B[e]P	B[a]P	Ind	D[a,h]A	B[g,h,i]P
IS1	1.04E+05	1.49E+03	3.68E+03	1.08E+03	6.80E+02	1.31E+03	2.73E+02	1.97E+03
IS2	1.06E+05	5.88E+02	2.82E+03	4.13E+02	2.37E+02	3.10E+02	1.12E+02	4.16E+02
IS3	7.20E+04	1.10E+03	2.73E+03	6.66E+02	3.19E+02	5.60E+02	1.47E+02	1.04E+03
IS4	2.77E+03	2.41E+03	1.23E+03	1.46E+03	8.04E+02	1.42E+03	5.49E+02	2.54E+03
IS5	1.11E+03	1.01E+03	1.35E+03	8.26E+02	4.30E+02	6.93E+02	1.64E+02	1.22E+03
IS6	6.77E+02	5.38E+02	6.35E+02	3.64E+02	3.03E+02	3.32E+02	5.71E+01	5.91E+02
IS7	2.54E+05	3.88E+02	4.63E+03	2.80E+02	9.68E+01	2.55E+02	2.85E+01	3.97E+02
IS8	4.54E+05	1.15E+03	8.94E+03	1.33E+03	5.50E+02	7.93E+02	2.00E+02	1.45E+03
IS9	2.74E+05	1.43E+03	6.44E+03	9.33E+02	5.19E+02	8.05E+02	1.60E+02	1.13E+03
IS10	4.92E+05	1.13E+03	6.57E+03	7.70E+02	3.39E+02	6.36E+02	1.31E+02	1.10E+03
IS11	1.08E+03	1.10E+03	1.14E+03	8.86E+02	4.82E+02	7.94E+02	1.59E+02	1.62E+03
IS12	6.66E+02	5.91E+02	4.25E+02	3.31E+02	1.22E+02	3.17E+02	3.61E+01	4.89E+02
IS13	3.79E+02	3.37E+02	4.41E+02	1.69E+02	1.17E+02	1.58E+02	4.90E+01	2.23E+02
IS14	1.02E+03	5.92E+02	1.31E+03	3.22E+02	1.90E+02	2.20E+02	4.33E+01	3.23E+02
IS15	3.59E+02	3.27E+02	3.58E+02	2.13E+02	1.03E+02	1.65E+02	3.46E+01	2.69E+02
IS16	2.61E+02	3.88E+02	5.47E+02	1.89E+02	1.19E+02	1.49E+02	8.68E+01	2.12E+02
IS17	1.07E+03	8.48E+02	3.95E+02	4.83E+02	2.51E+02	5.01E+02	9.30E+01	7.55E+02
IS18	1.08E+03	3.23E+03	1.11E+03	1.29E+03	8.58E+02	1.76E+03	3.99E+02	2.17E+03
IS19	1.99E+02	1.67E+02	1.75E+02	8.85E+01	4.21E+01	8.67E+01	1.86E+01	1.10E+02
IS20	1.80E+02	2.81E+03	3.08E+03	1.66E+03	3.44E+02	1.26E+03	0.00E+00	1.40E+03
IS21	6.12E+02	1.43E+03	1.47E+03	9.45E+02	3.30E+02	7.27E+02	1.74E+02	1.49E+03
IS22	6.11E+02	2.70E+02	1.87E+02	1.61E+02	9.69E+01	1.76E+02	1.94E+02	2.66E+02
IS23	1.67E+02	3.12E+04	2.12E+04	1.75E+04	1.42E+03	1.35E+04	2.25E+03	1.11E+04
IS24	3.45E+03	2.20E+03	1.70E+03	1.21E+03	6.92E+02	1.20E+03	3.53E+02	1.83E+03
IS25	1.64E+02	2.45E+04	6.05E+04	1.32E+04	1.51E+03	8.87E+03	2.48E+03	8.50E+03
IS26	4.56E+02	2.08E+04	2.54E+04	1.62E+04	7.19E+03	1.57E+04	4.75E+03	2.83E+04
IS27	5.66E+01	4.35E+01	3.85E+01	2.17E+01	5.85E+00	1.17E+01	0.00E+00	1.72E+01
IS28	1.26E+04	6.53E+03	7.57E+02	3.93E+03	3.14E+03	3.90E+03	8.62E+02	4.87E+03
IS29	1.79E+03	1.78E+04	6.86E+03	1.02E+04	3.55E+03	8.24E+03	2.04E+03	8.79E+03
IS30	3.23E+02	3.06E+02	2.68E+02	1.33E+02	7.74E+01	1.03E+02	0.00E+00	1.46E+02
AB1	4.23E+05	4.28E+05	1.69E+05	2.68E+05	2.95E+05	1.88E+05	6.63E+04	1.85E+05
AB2	5.24E+03	5.31E+03	1.54E+03	3.19E+03	2.54E+03	2.60E+03	7.63E+02	3.45E+03
AB3	2.04E+03	6.12E+03	1.64E+03	3.52E+03	2.21E+03	2.94E+03	7.64E+02	3.57E+03
AB4	8.32E+03	7.76E+03	2.10E+03	7.16E+03	3.99E+03	3.59E+03	9.19E+02	6.44E+03
AB5	4.02E+04	3.07E+04	9.68E+03	1.91E+04	1.68E+04	1.22E+04	3.47E+03	1.52E+04
AB6	1.90E+03	1.50E+03	4.45E+02	1.19E+03	6.50E+02	7.85E+02	2.47E+02	1.28E+03
AB7	2.14E+03	2.75E+03	4.00E+03	1.61E+03	1.38E+03	1.21E+03	3.15E+02	1.68E+03
AB8	6.20E+03	5.79E+03	7.98E+03	4.09E+03	2.46E+03	3.15E+03	8.25E+02	5.06E+03
AB9	3.21E+03	2.03E+05	5.71E+04	1.26E+05	5.76E+04	9.84E+04	3.09E+04	1.27E+05
AB10	2.35E+04	3.32E+04	1.25E+04	2.01E+04	1.52E+04	1.79E+04	4.84E+03	2.28E+04
TA1	2.31E+03	2.59E+03	8.14E+02	1.53E+03	1.19E+03	1.27E+03	2.94E+02	1.67E+03
TA2	2.75E+04	3.80E+04	1.17E+04	2.23E+04	1.95E+04	2.08E+04	5.38E+03	2.45E+04
TA3	2.75E+04	3.80E+04	1.17E+04	2.23E+04	1.95E+04	2.08E+04	5.38E+03	2.45E+04
TA4	1.05E+04	1.18E+04	1.91E+03	7.64E+03	4.96E+03	5.82E+03	1.44E+03	7.27E+03
TA5	3.85E+04	1.43E+05	4.54E+04	8.64E+04	6.60E+04	8.42E+04	2.45E+04	1.01E+05
SA-TOP	2.86E+03	4.34E+03	1.34E+03	2.88E+03	2.57E+03	2.50E+03	6.34E+02	3.57E+03
SA-10	4.02E+03	3.67E+03	1.18E+03	2.26E+03	1.86E+03	2.07E+03	5.67E+02	2.87E+03
SA-20	1.68E+03	3.11E+03	1.28E+03	2.03E+03	1.46E+03	1.77E+03	3.97E+02	2.60E+03
SA-30	2.76E+03	2.54E+03	6.17E+02	1.49E+03	1.06E+03	1.33E+03	3.41E+02	1.62E+03
SB-TOP	3.22E+02	4.88E+02	5.46E+02	2.47E+02	1.53E+02	2.51E+02	2.94E+01	3.56E+02
SB-10	3.84E+02	2.87E+02	6.95E+02	1.79E+02	9.04E+01	1.45E+02	1.20E+01	1.93E+02
SB-20	2.31E+02	5.81E+02	9.33E+02	3.19E+02	1.40E+02	2.48E+02	1.41E+01	3.44E+02
SB-30	1.90E+02	1.63E+02	2.45E+02	1.02E+02	4.11E+01	8.70E+01	1.39E+01	1.08E+02
SC-TOP	1.80E+03	2.44E+03	5.18E+02	1.47E+03	1.16E+03	1.50E+03	3.65E+02	2.08E+03
SC-10	3.57E+03	2.01E+04	2.52E+03	1.20E+04	8.83E+03	1.17E+04	2.96E+03	1.44E+04
SC-20	2.80E+02	1.47E+02	1.78E+02	7.68E+01	1.31E+01	3.15E+01	8.21E+01	4.81E+01
SC-30	2.07E+02	1.71E+02	1.43E+02	1.00E+02	1.84E+01	5.73E+01	3.93E+01	9.52E+01
SD-TOP	6.49E+02	7.49E+02	3.13E+02	4.43E+02	2.57E+02	4.10E+02	9.50E+01	4.94E+02
SD-10	1.08E+03	9.23E+02	3.82E+02	5.34E+02	2.94E+02	4.58E+02	9.45E+01	5.41E+02
SD-20	1.34E+03	2.46E+03	4.41E+02	8.65E+02	1.24E+03	9.87E+02	1.37E+02	1.23E+03
SD-30	3.72E+02	7.49E+02	4.97E+02	4.72E+02	2.57E+02	3.66E+02	5.45E+01	5.16E+02
S1-TOP	3.20E+02	4.42E+02	4.80E+02	2.01E+02	1.72E+02	1.78E+02	2.24E+01	2.66E+02
S1-10	1.70E+02	2.02E+02	3.20E+02	1.03E+02	6.03E+01	8.29E+01	1.40E+01	1.20E+02
S1-20	2.88E+02	2.19E+02	2.59E+02	9.97E+01	7.53E+01	9.64E+01	4.85E+01	1.45E+02
S1-30	4.28E+02	4.68E+02	4.28E+02	2.20E+02	2.01E+02	2.07E+02	2.33E+01	2.88E+02
S2-TOP	9.35E+02	5.09E+02	8.33E+02	2.52E+02	9.83E+01	2.27E+02	5.75E+01	4.13E+02
S2-10	7.15E+02	5.53E+02	1.91E+02	3.02E+02	1.92E+02	2.65E+02	5.37E+01	4.33E+02
S2-20	5.05E+02	5.15E+02	1.59E+02	2.55E+02	1.82E+02	2.26E+02	3.42E+01	3.27E+02
S2-30	3.15E+02	1.48E+02	8.32E+01	7.32E+01	4.18E+01	5.59E+01	6.07E+00	9.82E+01
S3-TOP	1.97E+02	1.57E+02	2.73E+02	1.17E+02	6.44E+01	6.63E+01	0.00E+00	1.50E+02
S3-10	1.03E+02	5.28E+01	1.55E+02	3.56E+01	1.06E+01	1.40E+01	0.00E+00	4.32E+01
S3-20	9.30E+01	5.24E+01	2.11E+02	3.39E+01	1.35E+01	1.59E+01	0.00E+00	2.98E+01
S3-30	1.12E+02	8.19E+01	3.86E+02	5.34E+01	2.30E+01	2.13E+01	0.00E+00	9.38E+01
S4-TOP	1.26E+03	1.98E+03	8.21E+02	7.42E+02	6.83E+02	9.84E+02	1.76E+02	1.20E+03
S4-10	1.24E+03	2.53E+03	9.78E+02	9.87E+02	9.48E+02	1.31E+03	2.28E+02	1.62E+03
S4-20	8.29E+02	3.48E+03	2.21E+03	1.31E+03	1.19E+03	1.85E+03	3.59E+02	2.30E+03
S4-30	8.15E+02	1.49E+03	9.04E+02	5.39E+02	4.34E+02	7.03E+02	1.34E+02	8.41E+02
S5-TOP	1.43E+02	1.85E+02	2.26E+02	1.10E+02	2.35E+01	8.09E+01	0.00E+00	1.22E+02
S5-10	1.92E+03	1.94E+03	1.09E+03	7.27E+02	6.13E+02	8.10E+02	1.22E+02	1.02E+03
S5-20	1.38E+02	1.79E+02	2.19E+02	1.06E+02	2.28E+01	7.84E+01	0.00E+00	1.19E+02
S5-30	1.21E+03	1.82E+03	1.16E+03	6.94E+02	5.94E+02	8.02E+02	1.25E+02	1.00E+03
S6-TOP	1.01E+03	1.72E+03	1.45E+03	6.96E+02	4.71E+02	7.64E+02	1.07E+02	9.83E+02
S6-10	1.74E+02	1.15E+02	2.31E+02	5.10E+01	1.56E+01	3.56E+01	0.00E+00	4.96E+01
S6-20	2.47E+02	1.45E+02	3.91E+02	6.83E+01	2.40E+01	5.10E+01	0.00E+00	8.79E+01
S6-30	1.37E+02	1.27E+02	2.81E+02	7.57E+01	2.94E+01	5.39E+01	0.00E+00	1.01E+02
S7-TOP	7.70E+02	1.00E+03	1.40E+03	5.42E+02	3.81E+02	5.40E+02	9.72E+01	7.23E+02
S7-10	5.43E+02	6.79E+02	1.21E+03	4.02E+02	2.92E+02	4.02E+02	7.41E+01	5.94E+02
S7-20	2.13E+03	7.43E+03	3.47E+03	4.23E+03	4.27E+03	3.31E+03	9.04E+02	4.06E+03
S7-30	2.56E+02	2.10E+02	5.33E+02	1.25E+02	6.97E+01	9.61E+01	1.31E+01	1.26E+02
S8-TOP	1.26E+03	8.43E+02	3.85E+02	4.99E+02	3.32E+02	4.34E+02	6.83E+01	5.97E+02
S8-10	1.47E+03	1.99E+03	5.46E+02	1.17E+03	8.00E+02	1.04E+03	2.14E+02	1.36E+03
S8-20	1.49E+03	1.76E+03	7.94E+02	1.08E+03	7.38E+02	8.90E+02	1.87E+02	1.18E+03
S8-30	1.57E+03	1.83E+03	1.02E+03	1.10E+03	7.71E+02	8.48E+02	1.72E+02	1.15E+03
S9-TOP	7.45E+02	8.31E+02	5.83E+02	5.25E+02	3.65E+02	4.28E+02	1.02E+02	6.38E+02
S9-10	5.70E+02	1.08E+03	7.51E+02	6.48E+02	4.14E+02	4.92E+02	1.53E+02	6.97E+02
S9-20	9.46E+02	1.05E+03	1.37E+03	4.97E+02	3.06E+02	4.05E+02	6.65E+01	6.00E+02
S9-30	1.04E+03	6.48E+02	4.18E+02	3.92E+02	2.68E+02	2.91E+02	5.94E+01	4.18E+02
S10-TOP	4.47E+02	5.47E+02	2.83E+02	3.41E+02	2.00E+02	2.59E+02	4.97E+01	3.14E+02
S10-20	1.34E+02	1.70E+02	5.54E+02	1.12E+02	6.14E+01	7.99E+01	0.00E+00	1.96E+02
S10-30	9.06E+01	1.94E+02	9.76E+02	1.16E+02	4.93E+01	6.43E+01	0.00E+00	9.76E+01
S11-TOP	2.04E+03	4.22E+05	1.50E+05	2.30E+05	1.16E+05	1.72E+05	6.06E+04	1.90E+05
S11-10	3.89E+02	2.20E+02	3.57E+02	1.27E+02	8.86E+01	9.12E+01	1.19E+01	1.42E+02
S11-20	2.32E+02	1.02E+02	3.42E+02	6.16E+01	2.70E+01	4.44E+01	0.00E+00	5.93E+01
S11-30	1.60E+02	1.01E+02	2.23E+02	4.66E+01	1.63E+01	2.17E+01	0.00E+00	3.19E+01
S12-TOP	2.70E+02	0.00E+00	0.00E+00	0.00E+00	0.00E+00	0.00E+00	0.00E+00	0.00E+00
S12-10	1.27E+03	9.96E+02	1.03E+02	7.01E+02	5.06E+02	5.60E+02	1.72E+02	7.83E+02
S12-20	2.12E+03	3.28E+02	8.03E+02	2.18E+02	7.82E+01	1.07E+02	2.46E+01	1.68E+02
S12-30	2.77E+02	3.32E+02	3.95E+02	2.03E+02	1.18E+02	1.44E+02	2.69E+01	2.01E+02
S13-TOP	3.49E+02	2.15E+03	4.18E+03	1.24E+03	6.14E+02	9.75E+02	8.17E+01	1.32E+03
S13-10	2.73E+02	3.37E+02	5.64E+02	2.25E+02	1.34E+02	1.35E+02	2.22E+01	2.20E+02
S13-20	1.43E+03	2.95E+03	3.03E+03	1.98E+03	1.17E+03	1.62E+03	2.74E+02	2.61E+03
S13-30	2.34E+02	1.56E+02	3.23E+02	9.88E+01	5.71E+01	5.40E+01	9.28E+00	8.30E+01
S14-TOP	5.95E+02	2.34E+02	2.73E+02	1.31E+02	7.62E+01	9.48E+01	1.35E+01	1.47E+02
S14-10	4.00E+02	1.90E+02	2.73E+02	1.17E+02	6.40E+01	8.13E+01	1.67E+01	1.18E+02
S14-20	2.33E+02	1.56E+02	3.22E+02	9.86E+01	5.70E+01	5.39E+01	9.26E+00	8.29E+01
S14-30	1.61E+02	7.57E+01	3.59E+02	4.06E+01	1.73E+01	2.10E+01	2.42E+01	2.73E+01
S15-TOP	1.39E+03	9.05E+02	2.79E+02	4.82E+02	4.17E+02	4.74E+02	1.14E+02	6.45E+02
S15-10	4.18E+02	3.54E+02	2.23E+02	2.09E+02	1.75E+02	1.96E+02	4.07E+01	3.14E+02
S15-20	8.21E+02	9.94E+02	2.28E+02	5.90E+02	5.92E+02	4.00E+02	1.20E+02	5.57E+02
S15-30	3.99E+02	8.60E+04	6.68E+04	5.59E+04	1.18E+04	3.67E+04	7.76E+03	4.59E+04

**Table 5:** PAH congener concentrations (pg g^−1^) of Phe, An, Fluo, Pyr, 11H-B[a]F, 11H-B[b]F, and B[a]A in terrestrial soils of King George Island, Antarctica.

	Phe	An	Fluo	Pyr	11H-B[a]F	11H-B[b]F	B[a]A
P1	2.01E+02	1.38E+04	2.35E+03	3.32E+03	3.99E+02	2.92E+02	7.42E+02
P2	5.31E+02	1.96E+04	4.22E+03	3.33E+03	2.35E+02	1.59E+02	1.44E+03
P3	4.12E+03	1.66E+02	4.42E+02	4.40E+01	2.08E+01	2.54E+01	2.89E+01
P4	9.75E+03	2.12E+02	7.03E+02	9.73E+02	8.16E+01	5.47E+01	3.58E+01
P5	5.91E+03	1.07E+02	8.99E+02	6.82E+02	8.15E+01	1.13E+02	9.42E+01
P6	1.26E+02	2.10E+03	8.74E+02	1.14E+03	2.05E+02	1.54E+02	8.81E+01
P7	3.30E+02	3.22E+03	5.88E+02	4.54E+02	5.71E+01	3.77E+01	2.75E+01
P8	2.33E+03	N.D.	1.84E+02	N.D.	N.D.	N.D.	N.D.
P9	N.D.	2.42E+03	3.66E+02	3.92E+02	3.99E+01	5.91E+01	1.98E+01
P10	N.D.	8.67E+03	9.31E+02	8.99E+02	7.41E+01	4.83E+01	N.D.
P11	3.88E+02	8.77E+03	1.13E+03	1.25E+03	N.D.	N.D.	3.81E+01

**Table 6:** PAH congener concentrations (pg g^−1^) of Chry, B[b]F, B[k]F, B[e]P, B[a]P, Ind, D[a,h]A, and B[g,h,i]P in terrestrial soils of King George Island, Antarctica.

	Chry	B[b]F	B[k]F	B[e]P	B[a]P	Ind	D[a,h]A	B[g,h,i]P
P1	4.03E+03	3.36E+01	1.05E+01	N.D.	2.10E+01	N.D.	N.D.	N.D.
P2	1.53E+03	N.D.	1.33E+03	N.D.	8.50E+02	N.D.	7.53E+01	N.D.
P3	1.60E+02	N.D.	3.80E+00	N.D.	N.D.	N.D.	N.D.	N.D.
P4	1.11E+02	N.D.	6.26E+01	4.38E+00	N.D.	N.D.	N.D.	N.D.
P5	8.43E+02	2.52E+01	1.22E+01	7.93E+00	5.32E+00	N.D.	N.D.	N.D.
P6	3.08E+02	N.D.	N.D.	N.D.	N.D.	N.D.	N.D.	N.D.
P7	9.94E+01	N.D.	N.D.	N.D.	N.D.	N.D.	N.D.	N.D.
P8	N.D.	N.D.	N.D.	N.D.	N.D.	N.D.	N.D.	N.D.
P9	1.05E+02	N.D.	N.D.	N.D.	N.D.	N.D.	N.D.	N.D.
P10	N.D.	N.D.	N.D.	N.D.	N.D.	N.D.	N.D.	N.D.
P11	6.37E+02	N.D.	N.D.	N.D.	N.D.	N.D.	N.D.	N.D.

## 2 Experimental design, materials and methods

### 2.1 Dataset area

**Fig. 1:**
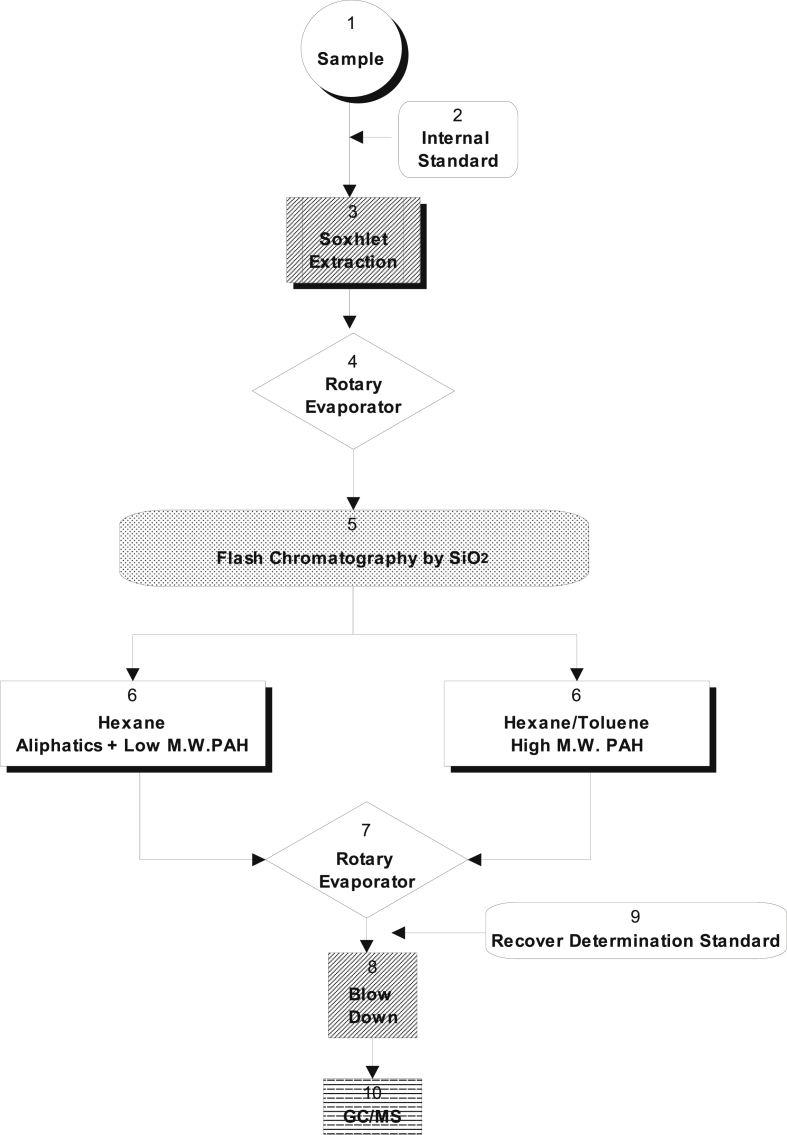
Analytical protocol of PAH congeners used in this study.

**Fig. 2:**
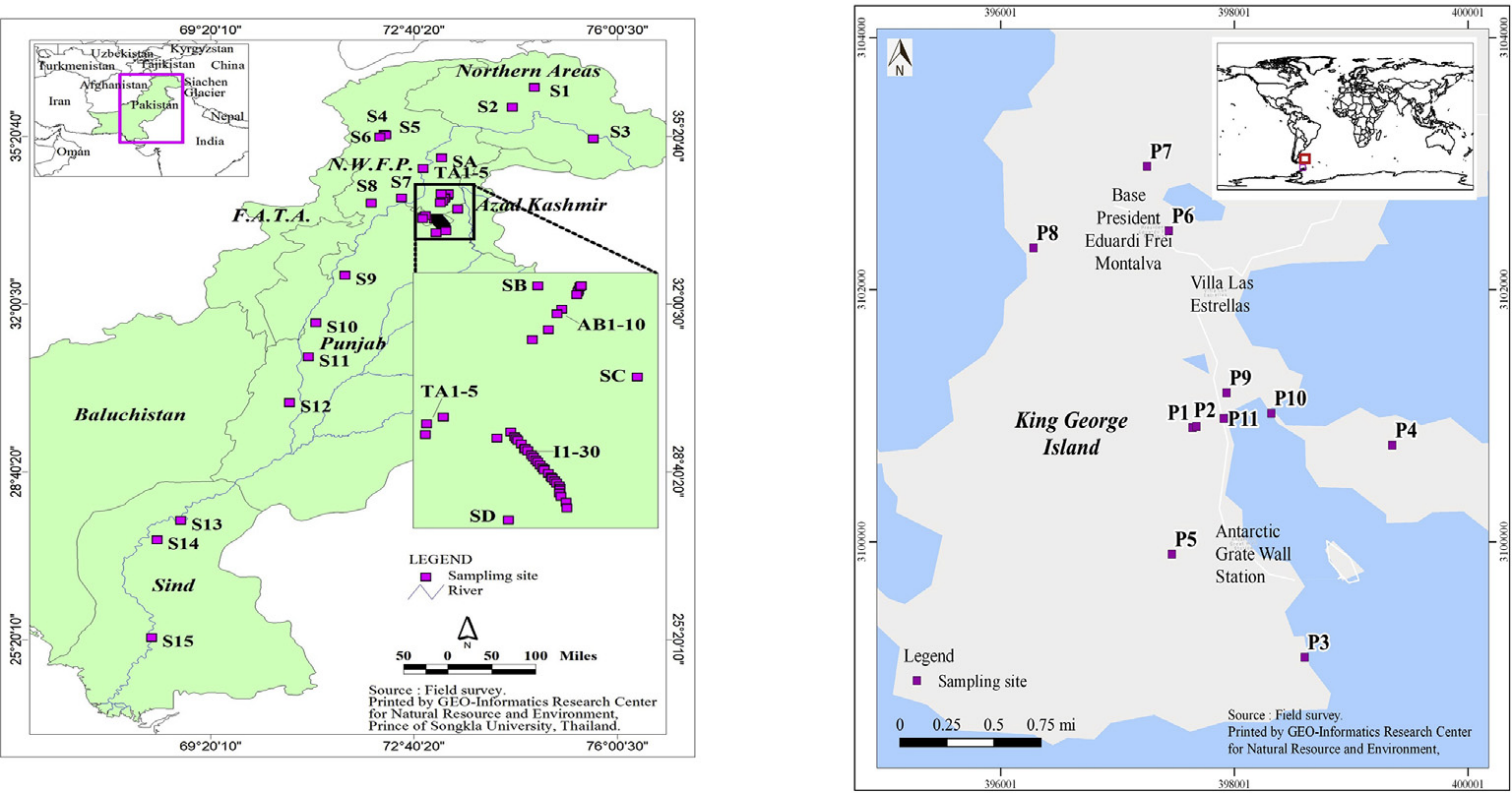
Sampling sites of terrestrial soils collected at Pakistan and King George Island, Antarctica.

All terrestrial soil samples were collected from different locations in Pakistan and King George Island (see Fig. 1, Fig. 2).

### 2.2 Sample collection and analytical procedures

In this study, about 0.3 kg of terrestrial soil samples from an area of 1 m^2^ at each sampling site was obtained by applying a shovel, which was stored in clean aluminium foil, situated in a glass bottle, and stored at −20 °C. After removing stones and shells, the samples were freeze-dried and sieved to <0.076 mm (200 mesh), and then stored at −20 °C until analysis. Details of the standard methods used for the soil sampling protocol can be found in previous publications [3], [4], [5]. Chemical analysis of PAH congeners are conducted in 2018 and described in Fig. 1. All details of GC-MS analysis are clearly explained in a previous study [7]. The fractionation/cleanup process followed the method reported by Gogou et al. (1996) [6]. After the extraction, the DCM solvent was concentrated to dryness by a combination of rotary evaporation and blowing under a gentle nitrogen stream. The concentrated extract is then diluted in 10 ml of n-hexane before application to the top of a disposable silica gel column. The extract was then fractionated into individual compound classes by flash chromatography on silica gel as follows: The concentrate was applied to the top of a 30 × 0.7 cm diameter column, containing 1.5 g of silica gel (activated at 150 °C for 3 h). Nitrogen pressure was used to in order to obtain a flow of 1.4 ml min^−1^ at the bottom of the column. The following solvents were used to elute the different compound classes: (1) 15 ml n-hexane (fraction 1, light molecular weight PAHs); (2) 15 ml toluene-n-hexane (5.6:9.4) (fraction 2, middle and heavy molecular weight PAHs). All solvents were of HPLC grade, purchased from Fisher Scientific. A mix of standard solutions of 13 native PAHs [Norwegian Standard (NS 9815: S-4008-100-T): phenanthrene (Phe), anthracene (An), fluoranthene (Fluo), pyrene (Pyr), benz[*a*]anthracene (B[*a*]A), chrysene (Chry), benzo[*b*]fluoranthene (B[*b*]F), benzo[*k*]fluoranthene (B[*k*]F), benzo[*a*]pyrene (B[*a*]P), benzo[*e*]pyrene (B[*e*]P), indeno[1,2,3-*c,d*]pyrene (Ind), dibenz[*a,h*]anthracene (D[*a,h*]A), and benzo[*g,h,i*]perylene (B[*g,h,i*]P), and a mix of recovery internal standard (IS) PAHs [*d*_12_-perylene (*d*_12_-Per) and *d*_10_-fluorene (*d*_10_-Fl)] were purchased from Chiron AS (Stiklestadveine 1, N-7041 Trondheim, Norway).
